# Supplementary material for: Using evidence when planning for trial recruitment: An international perspective from time-poor trialists
Source: PLoS One. 2019 Dec 10;14(12):e0226081. doi: 10.1371/journal.pone.0226081 (PMC6903711; doi:10.1371/journal.pone.0226081)
Supplement: S1 File — (DOCX) [file pone.0226081.s001.docx]

**Topic guide for Designers**

*Introduce yourself and give a brief outline of the project* – we’re doing a series of interviews with people involved in recruitment to trials, so that we can design future research, to make it more applicable to people actively doing recruitment at the moment.

- Check the participant understands what the interview entails (take copies of participant information leaflet for them to look over)
- Ask if there are any questions
- Ensure they are aware that confidentiality will be ensured at all times and their anonymity should direct quotes be used in publication

For telephone interviews:

- Request verbal consent and completion and return of paper copy of consent form in the post

For face to face interviews:

- Request written consent, ensure participant has completed form correctly (initials not ticks)

*Ok to turn on the recorder?*

Notes:

- Ask why – don’t assume
- Ask them first – don’t give examples unless they’re required
- Mode of delivery vs content of intervention
- Leave silences, don’t be tempted to fill them

Settling in questions

(Will be in variable orders depending on how conversation flows)

- Can you tell me a bit about your role and where you work?
- What is the part of your role that you enjoy most?
- And least?
- How long have you worked in clinical trials?
- What type(s) of trials have you worked on? How are they mainly funded?

Experiences with designing recruitment strategies for trials

- When I say ‘participant recruitment’ what does that mean to you?

*Clarify definition of participant recruitment*, the definition I tend to work from is: ‘the process of identifying potential participants, approaching them, telling them about the trial, inviting them to take part, and taking consent’.

- Can you tell me about your experience of designing recruitment strategies for trials?
  - *Explore disease indications/intervention types/population groups/multi-centre trials*
  - *Explore perceptions/experiences around the process of recruitment – challenging/straight-forward?*
- In your experience do you feel that recruitment is a similar process across all trials?
  - *Explore reasons behind their answer, why/how?*
- Which different groups do you work with when working on trials? Tell me about your experiences with that
  - *Explore impact of this on working environment/impact on recruitment*
  - This could be in relation to staff in their own unit and/or different groups e.g. other sites that they work with – either is useful so go with how the participant has interpreted the question first and then probe further for the alternative

Methods used to recruit

- What types of methods have you implemented to identify participants previously?
  - *Explore which worked/which didn’t work and reasons why*
- What types of methods have you thought about but not implemented?
  - *Explore reasons why*
- What methods to you use to approach participants?
  - *Explore which worked/which didn’t work and reasons why*
- What types of methods have you thought about but not used?
  - *Explore reasons why*
- In your experience do you find that recruitment methods are written into the grant application?
  - *Explore yes/no – if not why not? If so then why?*
- And protocol?
  - *Explore – if they’re in the grant application and in the protocol are they the same? Why? How do they differ?*
  - *If in the protocol do they tend to truly reflect the way the trial then recruits or do methods change throughout trial duration? How do methods change?*

Evidence base around recruitment

- How do you design recruitment strategies?
  - Looking for if they use evidence/resources/experience – *explore*
- What sort of resources do you know of to help you with designing recruitment strategies?
  - *Explore use of these if any known*
- What types of resource would you like to see available for you to use?
  - *Explore what these resource(s) would look like – what information would need to be included for them to be used?*
  - If participant doesn’t ‘get’ this give examples, *would it need to include numbers/a graph/text?*

*Finish off – revisit topics that haven’t been covered, ask if there is anything else they feel would be important for researchers to know; they’re the expert.*

Demographic data questions

- Stakeholder group (NHS/industry/academia):
- Gender:
- Age (years):
- Experience of working in trials (years):
- Interview duration:

*Thank participant for their time and contributions and switch off the recorder.*
